# Supplementary material for: Cumulative Weighing of Time in Intertemporal Tradeoffs
Source: J Exp Psychol Gen. 2016 Sep;145(9):1177–205. doi: 10.1037/xge0000198 (PMC4998108; doi:10.1037/xge0000198)
Supplement: Supplementary file 1 [file Scholten_Individuals.zip › plots/e30_p365_eg2_priors_and_posteriors.pdf]

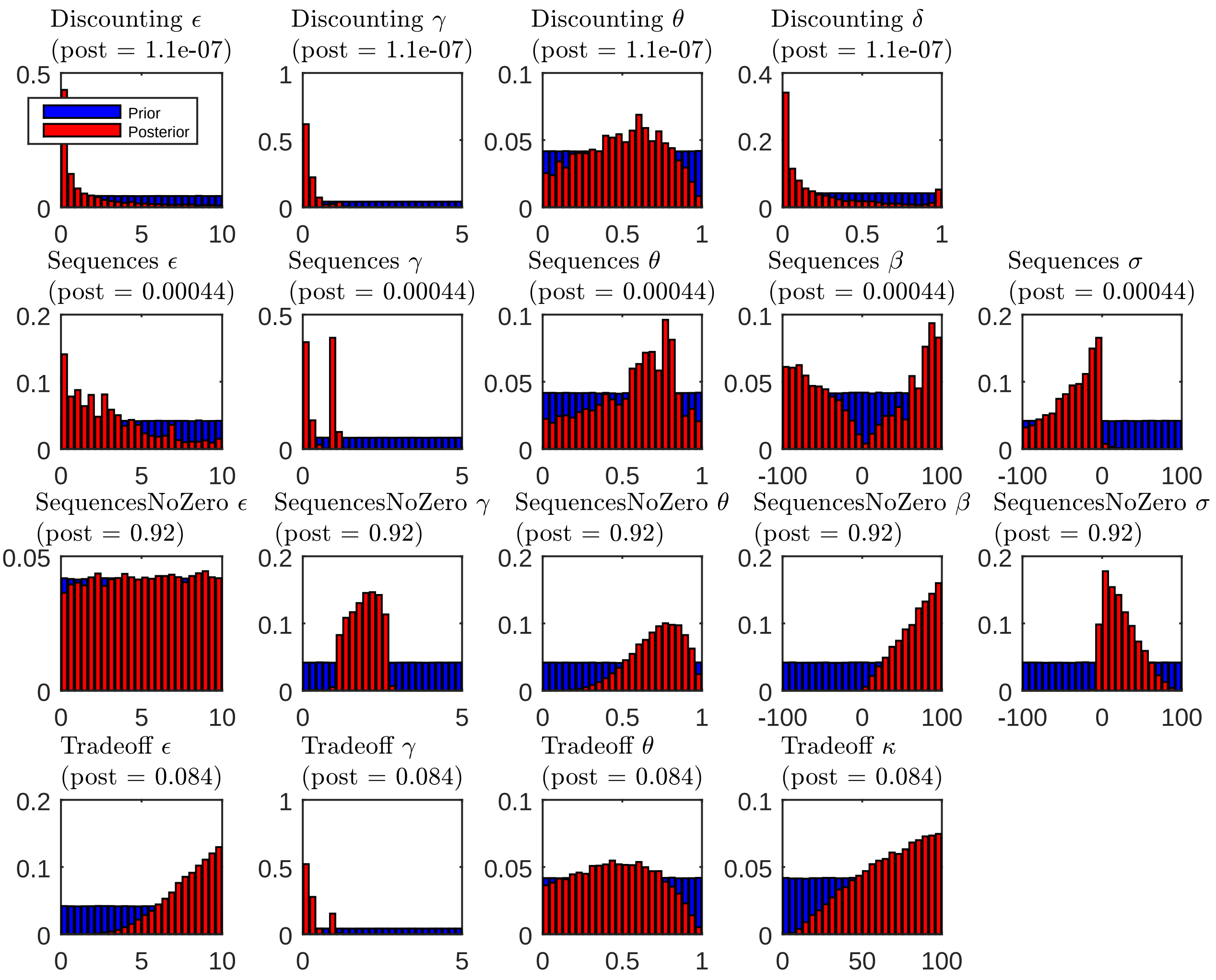
Discounting ϵ
(post = 1.1e-07)
Prior
Posterior
Discounting γ
(post = 1.1e-07)
Discounting θ
(post = 1.1e-07)
Discounting δ
(post = 1.1e-07)
Sequences ϵ
(post = 0.00044)
Sequences γ
(post = 0.00044)
Sequences θ
(post = 0.00044)
Sequences β
(post = 0.00044)
Sequences σ
(post = 0.00044)
SequencesNoZero ϵ
(post = 0.92)
SequencesNoZero γ
(post = 0.92)
SequencesNoZero θ
(post = 0.92)
SequencesNoZero β
(post = 0.92)
SequencesNoZero σ
(post = 0.92)
Tradeoff ϵ
(post = 0.084)
Tradeoff γ
(post = 0.084)
Tradeoff θ
(post = 0.084)
Tradeoff κ
(post = 0.084)
0 5 10
0 5
0 0.5 1
-100 0 100
0 50 100
0.5 1 0.1 0.05 0.4 0.2 0
